# Current Progress in Delineating the Roles of Pseudokinase TRIB1 in Controlling Human Diseases

**DOI:** 10.7150/jca.51627

**Published:** 2021-08-21

**Authors:** Xuelian Zhang, Bin Zhang, Chenyang Zhang, Guibo Sun, Xiaobo Sun

**Affiliations:** 1 Institute of Medicinal Plant Development, Peking Union Medical College and Chinese Academy of Medical Sciences, Beijing 100193, China.; 2 Key Laboratory of Bioactive Substances and Resources Utilization of Chinese Herbal Medicine, Ministry of Education, Beijing 100193, China.; 3 Beijing Key Laboratory of Innovative Drug Discovery of Traditional Chinese Medicine (Natural Medicine) and Translational Medicine, Beijing 100193, China.; 4 Key Laboratory of efficacy evaluation of Chinese Medicine against Glyeolipid Metabolism Disorder Disease, State Administration of Traditional Chinese Medicine, Beijing 100193, China.

**Keywords:** TRIB1, pseudokinase family, acute myeloid leukemia, prostate cancer, tumor drug resistance, cardiovascular disease

## Abstract

Tribbles homolog 1 (TRIB1) is a member of the tribbles family of pseudoprotein kinases and is widely expressed in numerous tissues, such as bone marrow, skeletal muscle, liver, heart, and adipose tissue. It is closely associated with acute myeloid leukemia, prostate cancer, and tumor drug resistance, and can interfere with the hematopoietic stem cell cycle, promote tumor cell proliferation, and inhibit apoptosis. Recent studies have shown that TRIB1 can regulate acute and chronic inflammation by affecting the secretion of inflammatory factors, which is closely related to the occurrence of hyperlipidemia and cardiovascular diseases. Given the important biological functions of TRIB1, the reviews published till now are not sufficiently comprehensive. Therefore, this paper reviews the progress in TRIB1 research aimed at exploring its roles in cancer, hyperlipidemia, and cardiovascular disease, and providing a theoretical basis for further studies on the biological roles of TRIB1.

## Introduction

Approximately 10% of the kinases in the human kinase group are named pseudokinases because they lack the necessary catalytic groups 1. Pseudokinases lack at least one motif in the catalytically related Val-Ala-Ile-Lys (VAIK) kinase domain; thus, only a small proportion of pseudokinases retain a low kinase activity 2-4. Although some pseudokinases do not possess kinase activity, they play a key role in regulating various cellular processes, such as the MAPK signaling pathway, actin polymerization, and the development of malignant tumor diseases 4-8.

Tribbles is a pseudoprotein kinase family, a branch of the human Ca^2+^/calmodulin-dependent protein kinase (CAMK) subfamily, with three homologous sequences (TRIB1-3) 1. According to sequence taxonomy, TRIB2 gene sequence is the most primitive in the tribbles family. TRIB2 orthologs can be traced back to the oldest metazoans, such as cnidaria and sponges, while the orthologs of TRIB1 and TRIB3 are limited in metazoan lineages 9. TRIB1-3 have different origins; however, they have the same central pseudokinase domain and C-terminal extension. Differences in the N-terminal extension have contributed to the structures of TRIB1, TRIB2, and TRIB3 not being conserved 10. The C-terminal extension of the Tribbles family can be combined with ubiquitin E3 ligase, allowing TRIB1-3 to target protein degradation for regulatory functions 4. The specific structure of the tribbles family allows the performance of specific biological functions, such as inhibiting embryo mitosis and regulating the cell cycle (TRIB1-3) 11, 12 , inducing myeloid leukemia (TRIB1-3) 13-15, inhibiting vascular smooth muscle migration (TRIB1 and TRIB3) 16, 17, and inducing Alzheimer's disease (TRIB3) 18.

**Figure 1:**
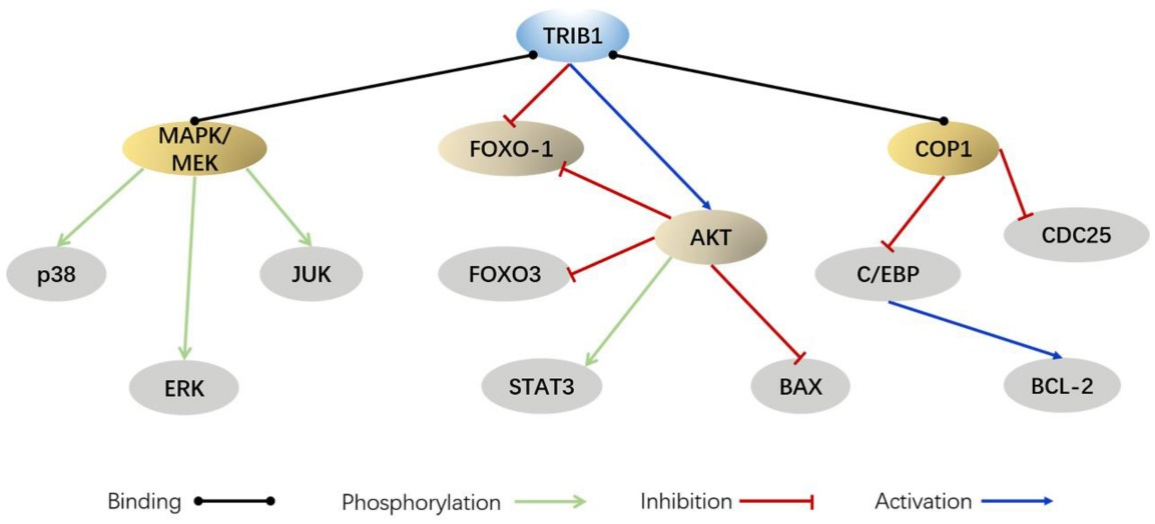
TRIB1 interaction pathways.

The human TRIB1 gene is located in the q24 and 13 regions of chromosome 8, the amino acid sequence of which is highly conserved. The amino-acid sequence homology between human and mouse TRIB1 is 97.5% 19. The N-terminus of TRIB1 protein is truncated and bent, making it incapable of forming the nucleotide-binding pocket of ATP 4. Thus, it lacks catalytic activity. Although TRIB1 is a pseudokinase, it has no catalytic activity and is widely expressed in various organ tissues such as the heart, liver, spleen, fat, and bone marrow, suggesting that it may possess other regulatory functions. TRIB1 has been reported to utilize its atypical pseudokinase domain as a substrate-regulated binding platform that ubiquitinates a target protein through an E3 ligase 20, 21. In addition, TRIB1 can also be used as a molecular scaffold that acts as a linker between the MEK/ERK pathway and the C/EBP family, thereby regulating cell proliferation and apoptosis 22 (Figure 1). Furthermore, TRIB1 has a wide range of biological effects and participates in material metabolism, inflammation, and endoplasmic reticulum stress response 23-25.

Although the relevant literature reviews the occurrence and participation of the tribbles family in leukemia and inflammation, these reviews remain limited for us to fully understand the essential roles of TRIB1 in the regulation of diseases such as cancer, inflammation, hyperlipidemia, and cardiovascular disease. Thus, we have summarized the progress made in experimental research on TRIB1 of the pseudokinase family. By reviewing the biological significance of TRIB1 in cancer, immunity, and lipid metabolism, this paper discusses the role of TRIB1 in the development of diseases and proposes new strategies for disease treatment.

## TRIB1 and cancer

Previous studies have found that TRIB1 is overexpressed in acute myeloid leukemia (AML), prostate cancer, and ovarian cancer and promotes tumor development. TRIB1 induces AML by promoting C/EBP ubiquitination 26. In addition, TRIB1 also promotes the proliferation of many types of tumors through FAK/SRC, ERK, and other pathways (Table 1). This suggests that TRIB1 can be used as a potential therapeutic target and general marker of cancer.

### TRIB1 and leukemia

AML is the most common subtype of leukemia in adults 27. Rothlisberger et al. found that TRIB1, which is located on the same chromosome as the leukemia-related gene MYC, also plays a key role in hematopoietic stem cell cycle regulation, metabolism, apoptosis, differentiation, cell adhesion, and tumorigenesis and participates in regulating hematopoietic homeostasis 28.

**Figure 2:**
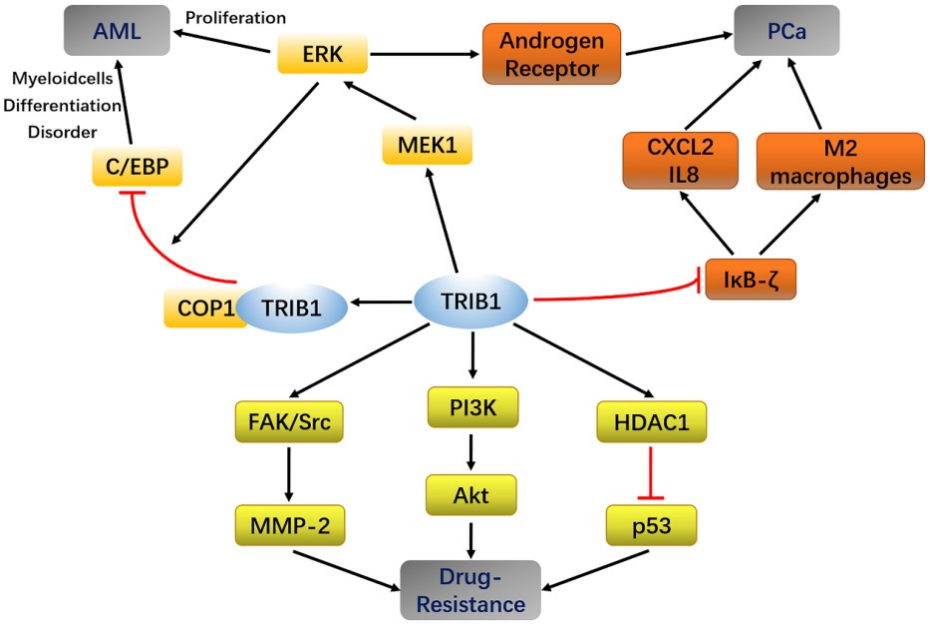
Summary of TRIB1 in Acute myeloid leukemia (AML), Prostate cancer (PCa), and drug resistance.

In AML, MYC and TRIB1 are significantly overexpressed compared to their expression in normal leukemia 28, 29. TRIB1 mainly depends on the MEK/ERK pathway and C/EBPα protein to influence AML occurrence and development. The highly conserved C-terminal ILLHPWF module of TRIB1 is used as a molecular scaffold to bind MEK1 to enhance ERK phosphorylation, thereby promoting cell proliferation and inhibiting apoptosis 22. In addition, the ILLHPWF module plays an important role in TRIB1-induced C/EBPα degradation. The ubiquitination/degradation of C/EBPα affects the cell cycle. During the hematopoietic process, wherein the hematopoietic stem cells mature into granulocyte-monocyte progenitor cells (GMPs), the protein levels of C/EBPα are significantly increased. Lack of C/EBPα, thus, not only leads to self-renewal disorders of hematopoietic stem cells but also inhibits GMP and granulocyte production, eventually inducing the occurrence of AML 30. The C-terminus of TRIB1 is used as a substrate-binding platform, which binds to constant photomorphogenesis protein 1 (COP1) and promotes ERK phosphorylation to bind to C/EBPα to degrade the target protein. Inhibition of ERK phosphorylation can reduce TRIB1-induced C/EBPα degradation, suggesting that the interaction between TRIB1 and MEK1 is necessary for the occurrence of TRIB1-induced leukemia 22, 31. Yoshino et al. also found evidence that TRIB1 regulates HOXA9-induced leukemia, suggesting that TRIB1 could be a potential therapeutic target for HOXA9-related AML. ERG silencing abrogated the growth advantage of TRIB1 overexpression in AML indicating that ERG is an important downstream target of TRIB1-HOXA9 axis 26. However, another study compared the transcriptome data of healthy controls and 285 AML patients and found that the expression of TRIB1 was significantly decreased in a small number of AML patients 32. Therefore, TRIB1 overexpression seems to be characteristic to certain cancer subtypes and may not be used as a general marker of cancer (Figure 2).

### TRIB1 and prostate cancer

Prostate cancer (PCa) is the most common non-skin cancer among men and the second leading cause of cancer death in men, with 1.1 million men diagnosed every year worldwide 33. As the baseline level of prostate-specific antigen (PSA) varies among different races, some cases cannot be accurately diagnosed 34. Thus, it is of great importance to identify novel genetic markers of PCa to achieve early and accurate diagnosis of PCa. Increasing evidence has shown that TRIB1 can directly or indirectly regulate the development of PCa through the androgen receptor (AR), NF-κB, and the endoplasmic reticulum partner glucose-regulated protein 78 kDa (GRP78) 35-37. Therefore, it may serve as a healing factor.

Moya et al. studied TRIB1 expression in clinically malignant PCa and normal tissues. They observed that the mRNA and protein levels of TRIB1 were increased in PCa tissues by more than three-fold 36, 38. RNA sequencing showed that the short tandem repeats of TRIB1 were elevated in PCa compared to those in the adjacent normal tissue cells, suggesting that TRIB1 is closely associated with the occurrence and development of PCa and could be a potential predictor of PCa healing 39. In addition to its potential as a diagnostic marker for PCa, studies have found that TRIB1 could also serve as a new target for treating PCa. TRIB1 can directly regulate ARs that signal through ERK phosphorylation, thereby driving the onset and progression of PCa 35. It can also suppress nuclear factor kappa B (NF-κB) activity by regulating IκB expression, indirectly affecting the development of PCa 37, 40. Deletion of the tumor suppressor *Pten* in the prostate epithelium leads to PCa. Shahrouzi et al. found that the expression of TRIB1 in the prostate of mice with PCa (*Pten^pc-/-^*) was higher than in wild-type controls (*Pten^pc+/+^*). They then developed a Cre-dependent TRIB1 transgenic mouse model, in which the incidence of PCa was increased in *Pten^pc+/-^* mice from 16.7% to 50% 41. Additionally, TRIB1 participates in the endoplasmic reticulum pathway and is required for the expression of the endoplasmic reticulum partner GRP78, which is closely related to the occurrence of prostate tumors 36.

At present, experiments have shown that miR-224 can exert a partial tumor inhibitory effect in PCa by targeting TRIB1 42. TRIB1 inhibits IKB-zeta in prostate cancer and promotes macrophages to the M2 phenotype 43. These findings reveal that the abnormal expression of TRIB1 may promote prostate cancer progression, thus making it a new potential biomarker for PCa diagnosis and prognostic evaluation (Figure 1).

## TRIB1 and tumor drug resistance

Chemotherapy is an important method for treating malignant tumors, including those of AML and PCa. With the extension of treatment time, the suppressive effects of some drugs on tumor progression gradually weaken, although they have significant curative effects in the early stages of cancer. The strong drug resistance of tumor cells causes failure in chemotherapy. In addition to being involved in cancers such as AML, PCa, and liver cancer, TRIB1 can also regulate tumor cell activity through pathways such as PI3K/AKT, HDAC1/p53 and C/EBPα, thereby affecting the resistance of tumor cells (Figure 1).

Cisplatin is commonly used in the clinical treatment of non-small cell lung cancer. Recent studies have shown that cisplatin can induce an increase in TRIB1 protein expression with an increase in treatment time. Increased TRIB1 subsequently activates the PI3K/AKT pathway and promotes tumor cell proliferation, migration, and invasion, and treatment of resistance, eventually leading to the accumulation of cancer stem cells and the generation of multidrug resistance 44, 45. Similarly, Wan et al. found that K562 cells promoted cell viability and increased adriamycin resistance by upregulating miR-103 expression. Overexpression of miR-103 significantly increased the expression of TRIB1, while the inhibitor of miR-103 restored the drug sensitivity of K562 cells through the regulation of COP1, TRIB1, and PI3K/AKT pathways 46.

The JAK-STAT signaling pathway is a cytokine-stimulated signal transduction pathway, which is involved in many important biological processes, such as cell proliferation, differentiation, apoptosis, and immune regulation. It has been found that TRIB1 plays a key role in macrophage differentiation through synergistic interactions with MAP/ERK and JAK/STAT pathways 47. TRIB1 can induce polarization of M2 macrophages 48, which play an important role in stimulating angiogenesis, tumor growth, and metastasis by secreting growth factors and cytokines 49. Chen et al. found that JAK1/2 pathway inhibition suppresses M2 polarization and overcomes myeloma resistance by reducing TRIB1 expression 50.

Furthermore, in triple-negative breast cancer, microarray technology was used to evaluate changes in gene expression patterns in paclitaxel-resistant Hs578T/Pax and MDA-MB-231/Pax cells. The changes in TRIB1 at the transcriptome and genomic levels were highly correlated with drug resistance. Although there was no further experimental explanation for this result, it also suggested the role of TRIB1 in drug resistance in triple-negative breast cancer 51.

In addition to regulating molecular pathways, Tang et al. found that TRIB1 can directly form a complex with phosphorylated histone deacetylase 1(HDAC1) to inhibit p53 expression in glioma cells. In contrast, glioma cells lacking TRIB1 exhibit enhanced responses to radiation-induced apoptosis 52. Inhibition of TRIB1 or HDAC1 has the potential to prevent or reduce radio resistance. Mechanistic research showed that TRIB1 enhanced HDAC1-mediated p53 deacetylation, decreased p53 binding to DNA, and reduced p53 tumor suppressor activity, suggesting that TRIB1 may be a new molecular target for cancer treatment 53 (Table 2).

## TRIB1 and inflammation

As is well known, TRIB1 affects the development of inflammation by affecting the differentiation of macrophages. Macrophages are generally divided into M1 and M2 subtypes 54, 55. M1 macrophages participate in the inflammatory response by secreting pro-inflammatory cytokines and chemokines, which target bacterial and viral infective host defense 56. M2 macrophages downregulate the immune response by secreting inhibitory cytokines IL-10 or TGF-β, which play an important role in parasite infection, tissue damage repair, and tumorigenesis development 57. Studies have found that TRIB1 molecules act as a linker protein, participating in the degradation of C/EBPα protein by interacting with COP1, thus resulting in the reduction of M2 macrophages in the spleen, liver, lung, peripheral blood, and bone marrow of TRIB1-deficient mice. High-fat-fed hematopoietic cell-specific TRIB1 knockout mice have increased triglyceride levels, insulin resistance, and proinflammatory cytokine expression 48.

In addition to M2 macrophages, TRIB1 also affects cytokine expression. TRIB1 can bind to pHDAC1, thereby reducing the inhibitory effect of HDAC1, activating nuclear factor of activated T-cells 2 (NFAT2), and promoting NFAT2-induced IL-2 transcription 58. TRIB1 knockout significantly reduces the activation of the IL-2 promoter, resulting in reduced IL-2 induction 58, 59. These results collectively indicate that TRIB1 is a positive regulator of IL-2 induction in activated T cells and a key transcription factor induced by IL-2. Furthermore, TRIB1 is a pro-inflammatory gene that regulates the production of TNF-α and controls the activity of NF-κB 60. In adipocytes, TRIB1 acts as a nuclear transcription coactivator of the NF-κB subunit RelA, thereby promoting the induction of pro-inflammatory cytokines in these cells 61. Studies have reported that the expression of TRIB1 is specifically upregulated during acute and chronic inflammation of white adipose tissue in mice. Deficiency of TRIB1 reduces cytokine gene expression in white adipocytes and prevents high-fat diet-induced obesity 61, 62.

Thus, TRIB1 participates in physiological and pathological reactions through immune cells such as macrophages, granulocytes, and regulatory T cells 63-65. Changes in TRIB1 levels are associated with the activation of NF-B and MAPK, and the production of inflammatory cytokines affects the development of inflammation, which in turn leads to autoimmune diseases, cancer, and obesity 66, 67.

## TRIB1 and hyperlipidemia

Genome-wide association analysis (GWAS) has released data over the past decade showing that more than 175 loci are strongly correlated with major circulating lipid levels. Most of these genes are specifically related to one or two lipids, while some genes, such as SUGP1, ZPR1, TRIB1, HERPUD1, and FADS1, are associated with all circulating lipid levels 68. Further research confirms that the TRIB1 gene is closely associated with TG and LDL-C levels, an increase in which may increase the risk of coronary artery disease 69.

Current studies on TRIB1 and lipids have mainly focused on blood lipids and liver fat. It has been found that an overexpression of TRIB1 in mouse liver leads to a decrease in plasma cholesterol and TG levels and a reduction in VLDL secretion 70, 71. TRIB1 can cooperate with SAP18 and mSin3A to activate the transcription of microsomal triglyceride transporter protein (MTTP) 72. Therefore, knocking out TRIB1 in human liver cells reduces the expression of MTTP and APOB 72-74. APOB protein is the main apolipoprotein component of VLDL and LDL. TRIB1 affects blood lipid levels by controlling the metabolism of lipid transporters in mice. In addition, TRIB1 is involved in lipogenesis. Carbohydrate response element binding protein (ChREBP) is a glucose-sensitive molecule involved in hepatic lipogenesis 75. Overexpression of TRIB1 can downregulated ChREBP mRNA and protein levels, thereby affecting adipogenesis 76. Meanwhile, TRIB1 liver-specific knockout mice showed increased VLDL production and increased plasma TG and TC levels 73. Moreover, the knockout of TRIB1 increased the expression of liver C/EBPα protein, thereby promoting the production and accumulation of fat in liver cells. Increased liver lipid deposition can lead to liver cell damage, which increases serum transaminase levels, liver steatosis, and non-alcoholic fatty liver disease 70, 77.

Studies have shown that mice with circadian disturbances or lack of circadian rhythm genes exhibit altered triglyceride and cholesterol metabolism, which then progress to diabetes, obesity, and metabolic syndrome 78, 79. Similar research suggests that sleep homeostasis plays a role in regulating lipid metabolism. Although the circadian rhythm of TRIB1 is weak in mice, it plays an important role in restoring sleep homeostasis after sleep restriction 80, 81. In the hyperlipidemia model, with the disruption of the PCSK9/LDL receptor regulation axis caused by the dark and light cycle disorder and the liver BMAL1 rhythm gene disorder, the overexpression of TRIB1 restored the plasma PCSK9 level, increased the expression of LDL receptor protein, and restored plasma cholesterol homeostasis in mice lacking the liver clock gene 81. In addition to restoring circadian rhythm through the PCSK9/LDL receptor regulation axis, we found that COP1 binding to TRIB1 also showed rhythmicity. COP1 is a highly conserved E3 ubiquitin ligase that shuttles between the nucleus and the cytoplasm 82. In plants, COP1 localization is controlled by light and exhibits a light-dark rhythm 83. In mammals, TRIB1 can mimic the light regulation of COP1 localization in plants. TRIB1 blocks the nuclear export of COP1 by disrupting intramolecular interactions within COP1 84. These sets of experimental evidence suggest that TRIB1 may be able to restore obesity and hyperlipidemia caused by circadian rhythm disorders by regulating the discipline gene. This part of the mechanism remains to be elucidated.

In addition to blood lipids and liver lipids, some studies have shown that TRIB1-deficient mice have adipose tissue metabolism disorders, reduced adipose tissue, and thus, the area of fat cells becomes smaller. Concurrently, the number of macrophages in the adipose tissue of TRIB1-deficient mice is also less than that in wild-type mice 48. In the case of a high-fat diet, TRIB1-deficient mice exhibit high levels of lipids, cholesterol, blood glucose, and insulin. Another review reported that the expression of TRIB1 is specifically upregulated during acute and chronic inflammation of WAT in mice. TRIB1 knockout reduces the expression of cytokine genes in white adipocytes and prevents high-fat diet-induced obesity 61. All lipid metabolism disorders caused by TRIB1 observed in these studies are discussed from the perspective of immune inflammation, and the relationship between TRIB1 and fat development remains to be explored.

## TRIB1 and cardiovascular disease

The regulatory effects of TRIB1 on circulating lipids and immune cells suggest that TRIB1 is associated with the development of cardiovascular diseases. Atherosclerosis is a chronic cardiovascular disease. Vascular wall damage plays an important role in the development of atherosclerotic plaques. Local abnormalities of vascular walls cause endothelial cell dysfunction or cell death, which leads to the production and release of a variety of inflammatory cytokines and chemokines 85. Monocytes are then recruited to the lesion site where they differentiate into macrophages and are transformed into foam cells by ingesting oxidized low-density lipoprotein (ox-LDL) particles to form vascular plaques 86.

A GWAS has shown that the TRIB1 gene is associated with elevated triglyceride levels. Elevated triglyceride levels represent an increased risk of cardiovascular diseases 87, 88. In the development of atherosclerosis, M2 macrophages are gradually replaced by M1 inflammatory macrophages, while TRIB1 is associated with the differentiation of M2 macrophages that maintains homeostasis and repair of damaged tissue 48, 89, 90. TRIB1 knockout leads to metabolic disorders and cardiovascular disease by affecting the repair of M2 macrophages. In addition, under pathogenic conditions, mitogen-activated protein kinase (MAPK) is associated with inflammatory factor-mediated migration and proliferation of vascular smooth muscle cells (VSMCs) 91, 92. TRIB1 regulates the activity of the MAPK cascade by binding to MAPKK (MAPK kinase). Recent studies have shown that TRIB1 is a negative regulator of MAPKK4 activation of c-Jun N-terminal kinase in vascular smooth muscle cells, which can reduce the chemotaxis of inflammatory factors and inhibit the migration and proliferation of vascular smooth muscle cells 93, 94.

The above studies show that the expression of TRIB1 can reduce the risk of cardiovascular disease through reducing triglyceride levels, maintaining steady-state phagocytosis and percentages of M2 macrophages, and inhibiting the chemotaxis of inflammatory factors and cardiovascular reconstruction. We speculate that TRIB1 may be a favorable factor in cardiovascular diseases. Interestingly, the latest experimental data from Johnston et al. showed that a decrease in the expression of myeloid-specific TRIB1 can inhibit the formation of early atherosclerosis, and an increase in the level of TRIB1 transcription can cause atherosclerosis 95. It has been reported that TRIB1 increases the accumulation of macrophage lipids, and the expression of key receptors (OLR1) promotes the uptake of oxidized low-density lipoproteins and forms lipid-rich foam cells. These inconsistent results may indicate that drug therapy with specific silencing of TRIB1 expression in macrophages may need to be adopted in the early stages of atherosclerosis.

## Conclusion

The regulatory role of TRIB1 as a pseudokinase protein in tumor development is extremely important. In recent years, in addition to cancers and cardiovascular diseases, chronic diseases such as chronic low-grade inflammation and lipid metabolism disorders have also attracted great attention. TRIB1 is also involved in macrophage polarization, inflammatory factor regulation, and fat synthesis, linking inflammation and cardiovascular disease. However, this is only based on the speculation of the current literature and the relationship between TRIB1. Hyperlipidemia, inflammation, and cardiovascular diseases require further research.

In summary, TRIB1 participates in multiple signal transduction pathways in humans and can show synergistic or completely antagonistic effects in different pathways. TRIB1 is associated with tumorigenesis in acute leukemia and prostate cancer. TRIB1 is also involved in a series of non-neoplastic diseases, including metabolic disorders, cardiovascular diseases, and autoimmune diseases. TRIB1 plays a particularly important role in the differentiation and development of M2-like macrophages inherent in tissues, and thus affects the body's inflammation and development, and the adipose tissue metabolism. The above studies have enriched our knowledge of macrophages. An understanding of the mechanism of cell differentiation and development also provides new ideas for the treatment of related diseases.

## Figures and Tables

**Table 1:** Cellular and molecular mechanisms of TRIB1 on cancer

Cancer	Mechanisms	References
Prostate cancer	TRIB1 promoted CXCL and IL8 secretion by inhibiting IKB-zeta expression and induced tumor growth; TRIB1 supported the occurrence of prostate cancer and the survival of tumor cells through upregulation of GRP78 in prostate cancer.	36, 43, 46
Hepatocellular carcinoma	TRIB1 promoted the survival of HCC cells with down-regulation of p53, and induced migration, invasion and epithelial mesenchymal transition of HCC cells.	45, 96
Acute myeloid leukemia	The binding of TRIB1 with MEK1 enhanced ERK phosphorylation and induced the downregulation of C/EBP α protein expression; COP1 and TRIB1 form oncoprotein complexes, which promote the degradation of C/EBP α; TRIB1/HOXA9 axis accelerates leukemia by up regulating the expression of ERG.	21,26,46
Non-small cell lung cancer	In NSCLC, the interaction between TRIB1 and HDAC mediates the inactivation of p53. In addition, TRIB1 also participates in the abnormal activation of PI3K/Akt pathway and promotes the proliferation of cancer cells.	44, 45
Breast invasive ductal cancer	TRIB1 improves cancer-associated fibroblast activity.	97
Colorectal cancer	TRIB1 activates MMP-2 through FAK / Src and ERK pathways and promotes colorectal cancer cell migration.	98, 99
Human glioma	TRIB1 formed a complex with pHDAC1 to inhibit the expression of p53 in glioma cells and increased the radioresistance of tumor cells.	52

**Table 2:** TRIB1 and tumor drug resistance

Pathway	Cancer	Biological consequence	References
PI3K/AKT	Non-small cell lung cancer; Acute myeloid leukemia	Over activation and up regulation of PI3K/Akt signaling pathway promoted tumor proliferation, migration, invasion and drug resistance.	44, 46
HDAC1/p53	Non-small cell lung cancer; Human glioma; Human breast cancer	TRIB1 enhanced histone deacety lase 1 (HDAC1)-mediated p53 deacetylation and decreased tumor suppressor activity of p53.	45, 52, 53
JAK1/2	Myeloma	JAK/STAT pathway may mediate M2 polarization by increasing TRIB1, and TRIB1 induces polarization of M2 macrophages, promotes tumor growth and metastasis, and enhances drug resistance.	50
Erk1/2	Triple negative breast cancer	IL-6, CXCL8, VEGFA, EGR1, PTGS2 and TRIB1 upregulate and mediate drug resistance in the treatment of breast cancer with paclitaxel.	51

## Abbreviations

TRIB1/2/3: Tribbles homolog 1/2/3
MAPK: Mitogen-activated protein kinase
MAPKK: MAPK kinase
CAMK: Calcium/calmodulin-dependent protein kinases
ATP: Adenosine triphosphate
MEK: Mitogen-activated protein kinase kinase
ERK: Extracellular regulated protein kinases
C/EBP: CCAAT/enhancer binding protein
AML: Acute myeloid leukemia
GMP: Granulocyte-monocyte progenitor cells
COP1: Constant photomorphogenesis protein 1
PCa: Prostate cancer
PSA: Prostate-specific antigen
NF-κB: Nuclear factor kappa-B
GRP78: Glucose regulated protein 78 kD
PI3K/AKT: phosphatidylinositol 3 kinase/protein kinase B
HDAC1: Histone deacetylase 1
TGF-β: Transforming growth factor-β
NFAT2: Nuclear factor 2
TNF-α: Tumor necrosis factor-α
GWAS: Genome-wide association analysis
MTTP: Microsomal triglyceride transporter protein
ChREBP: Carbohydrate response element binding protein
NAFLD: Non-alcoholic fatty liver disease
PCSK9: Proprotein convertase subtilisin kexin type 9
WAT: White adipose tissue
VSMC: Vascular smooth muscle cells

## References

[1] Manning G, Whyte DB, Martinez R, Hunter T, Sudarsanam S (2002). The protein kinase complement of the human genome. Science.

[2] Eyers PA, Murphy JM (2013). Dawn of the dead: protein pseudokinases signal new adventures in cell biology. Biochemical Society transactions.

[3] Murphy JM, Zhang Q, Young SN, Reese ML, Bailey FP, Eyers PA (2014). A robust methodology to subclassify pseudokinases based on their nucleotide-binding properties. The Biochemical journal.

[4] Murphy JM, Nakatani Y, Jamieson SA, Dai W, Lucet IS, Mace PD (2015). Molecular Mechanism of CCAAT-Enhancer Binding Protein Recruitment by the TRIB1 Pseudokinase. Structure.

[5] Scheeff ED, Eswaran J, Bunkoczi G, Knapp S, Manning G (2009). Structure of the pseudokinase VRK3 reveals a degraded catalytic site, a highly conserved kinase fold, and a putative regulatory binding site. Structure.

[6] Fukuda K, Gupta S, Chen K, Wu C, Qin J (2009). The pseudoactive site of ILK is essential for its binding to alpha-Parvin and localization to focal adhesions. Molecular cell.

[7] Stefely JA, Reidenbach AG, Ulbrich A, Oruganty K, Floyd BJ, Jochem A (2015). Mitochondrial ADCK3 employs an atypical protein kinase-like fold to enable coenzyme Q biosynthesis. Molecular cell.

[8] Toms AV, Deshpande A, McNally R, Jeong Y, Rogers JM, Kim CU (2013). Structure of a pseudokinase-domain switch that controls oncogenic activation of Jak kinases. Nature structural & molecular biology.

[9] Eyers PA, Keeshan K, Kannan N (2017). Tribbles in the 21st Century: The Evolving Roles of Tribbles Pseudokinases in Biology and Disease. Trends Cell Biol.

[10] Kiss-Toth E, Wyllie DH, Holland K, Marsden L, Jozsa V, Oxley KM (2006). Functional mapping and identification of novel regulators for the Toll/Interleukin-1 signalling network by transcription expression cloning. Cell Signal.

[11] Mata J, Curado S, Ephrussi A, Rorth P (2000). Tribbles coordinates mitosis and morphogenesis in Drosophila by regulating string/CDC25 proteolysis. Cell.

[12] Seher TC, Leptin M (2000). Tribbles, a cell-cycle brake that coordinates proliferation and morphogenesis during Drosophila gastrulation. Curr Biol.

[13] Nakamura T (2015). The role of Trib1 in myeloid leukaemogenesis and differentiation. Biochemical Society transactions.

[14] Salome M, Magee A, Yalla K, Chaudhury S, Sarrou E, Carmody RJ (2018). A Trib2-p38 axis controls myeloid leukaemia cell cycle and stress response signalling. Cell death & disease.

[15] Li K, Wang F, Cao WB, Lv XX, Hua F, Cui B (2017). TRIB3 Promotes APL Progression through Stabilization of the Oncoprotein PML-RARalpha and Inhibition of p53-Mediated Senescence. Cancer cell.

[16] Sung HY, Guan H, Czibula A, King AR, Eder K, Heath E (2007). Human tribbles-1 controls proliferation and chemotaxis of smooth muscle cells via MAPK signaling pathways. The Journal of biological chemistry.

[17] Chan MC, Weisman AS, Kang H, Nguyen PH, Hickman T, Mecker SV (2011). The amiloride derivative phenamil attenuates pulmonary vascular remodeling by activating NFAT and the bone morphogenetic protein signaling pathway. Molecular and cellular biology.

[18] Lorenzi M, Altmann A, Gutman B, Wray S, Arber C, Hibar DP (2018). Susceptibility of brain atrophy to TRIB3 in Alzheimer's disease, evidence from functional prioritization in imaging genetics. Proc Natl Acad Sci U S A.

[19] Yokoyama T, Nakamura T (2011). Tribbles in disease: Signaling pathways important for cellular function and neoplastic transformation. Cancer Sci.

[20] Qi L, Heredia JE, Altarejos JY, Screaton R, Goebel N, Niessen S (2006). TRB3 links the E3 ubiquitin ligase COP1 to lipid metabolism. Science.

[21] Yoshida A, Kato JY, Nakamae I, Yoneda-Kato N (2013). COP1 targets C/EBPalpha for degradation and induces acute myeloid leukemia via Trib1. Blood.

[22] Yokoyama T, Kanno Y, Yamazaki Y, Takahara T, Miyata S, Nakamura T (2010). Trib1 links the MEK1/ERK pathway in myeloid leukemogenesis. Blood.

[23] Ishizuka Y, Nakayama K, Ogawa A, Makishima S, Boonvisut S, Hirao A (2014). TRIB1 downregulates hepatic lipogenesis and glycogenesis via multiple molecular interactions. Journal of molecular endocrinology.

[24] Brisard D, Chesnel F, Elis S, Desmarchais A, Sanchez-Lazo L, Chasles M (2014). Tribbles expression in cumulus cells is related to oocyte maturation and fatty acid metabolism. Journal of ovarian research.

[25] Arndt L, Dokas J, Gericke M, Kutzner CE, Muller S, Jeromin F (2018). Tribbles homolog 1 deficiency modulates function and polarization of murine bone marrow-derived macrophages. The Journal of biological chemistry.

[26] Yoshino S, Yokoyama T, Sunami Y, Takahara T, Nakamura A, Yamazaki Y (2020). Trib1 promotes acute myeloid leukemia progression by modulating the transcriptional programs of Hoxa9. Blood.

[27] Huang S, Zhang B, Fan W, Zhao Q, Yang L, Xin W (2019). Identification of prognostic genes in the acute myeloid leukemia microenvironment. Aging.

[28] Rothlisberger B, Heizmann M, Bargetzi MJ, Huber AR (2007). TRIB1 overexpression in acute myeloid leukemia. Cancer Genet Cytogenet.

[29] Hoffman B, Amanullah A, Shafarenko M, Liebermann DA (2002). The proto-oncogene c-myc in hematopoietic development and leukemogenesis. Oncogene.

[30] Friedman AD (2015). C/EBPalpha in normal and malignant myelopoiesis. Int J Hematol.

[31] Dedhia PH, Keeshan K, Uljon S, Xu L, Vega ME, Shestova O (2010). Differential ability of Tribbles family members to promote degradation of C/EBPalpha and induce acute myelogenous leukemia. Blood.

[32] Gilby DC, Sung HY, Winship PR, Goodeve AC, Reilly JT, Kiss-Toth E (2010). Tribbles-1 and -2 are tumour suppressors, down-regulated in human acute myeloid leukaemia. Immunol Lett.

[33] Campos C, Sotomayor P, Jerez D, Gonzalez J, Schmidt CB, Schmidt K (2018). Exercise and prostate cancer: From basic science to clinical applications. The Prostate.

[34] Lim J, Bhoo-Pathy N, Sothilingam S, Malek R, Sundram M, Hisham Bahadzor B (2014). Ethnicity is an independent determinant of age-specific PSA level: findings from a multiethnic Asian setting. PLoS One.

[35] Munkley J, Lafferty NP, Kalna G, Robson CN, Leung HY, Rajan P (2015). Androgen-regulation of the protein tyrosine phosphatase PTPRR activates ERK1/2 signalling in prostate cancer cells. BMC Cancer.

[36] Mashima T, Soma-Nagae T, Migita T, Kinoshita R, Iwamoto A, Yuasa T (2014). TRIB1 supports prostate tumorigenesis and tumor-propagating cell survival by regulation of endoplasmic reticulum chaperone expression. Cancer Res.

[37] Bornigen D, Tyekucheva S, Wang X, Rider JR, Lee GS, Mucci LA (2016). Computational Reconstruction of NFkappaB Pathway Interaction Mechanisms during Prostate Cancer. PLoS Comput Biol.

[38] Moya L, Lai J, Hoffman A, Srinivasan S, Panchadsaram J, Chambers S (2018). Association Analysis of a Microsatellite Repeat in the TRIB1 Gene With Prostate Cancer Risk, Aggressiveness and Survival. Front Genet.

[39] Lai J, Moya L, An J, Hoffman A, Srinivasan S, Panchadsaram J (2017). A microsatellite repeat in PCA3 long non-coding RNA is associated with prostate cancer risk and aggressiveness. Sci Rep.

[40] Gendelman R, Xing H, Mirzoeva OK, Sarde P, Curtis C, Feiler HS (2017). Bayesian Network Inference Modeling Identifies TRIB1 as a Novel Regulator of Cell-Cycle Progression and Survival in Cancer Cells. Cancer Res.

[41] Shahrouzi P, Astobiza I, Cortazar AR, Torrano V, Macchia A, Flores JM (2020). Genomic and Functional Regulation of TRIB1 Contributes to Prostate Cancer Pathogenesis. Cancers (Basel).

[42] Lin ZY, Huang YQ, Zhang YQ, Han ZD, He HC, Ling XH (2014). MicroRNA-224 inhibits progression of human prostate cancer by downregulating TRIB1. Int J Cancer.

[43] Liu ZZ, Han ZD, Liang YK, Chen JX, Wan S, Zhuo YJ (2019). TRIB1 induces macrophages to M2 phenotype by inhibiting IKB-zeta in prostate cancer. Cell Signal.

[44] De Marco C, Laudanna C, Rinaldo N, Oliveira DM, Ravo M, Weisz A (2017). Specific gene expression signatures induced by the multiple oncogenic alterations that occur within the PTEN/PI3K/AKT pathway in lung cancer. PLoS One.

[45] Wang L, Liu X, Ren Y, Zhang J, Chen J, Zhou W (2017). Cisplatin-enriching cancer stem cells confer multidrug resistance in non-small cell lung cancer via enhancing TRIB1/HDAC activity. Cell Death Dis.

[46] Wan L, Tian Y, Zhang R, Peng Z, Sun J, Zhang W (2018). MicroRNA-103 confers the resistance to long-treatment of adriamycin to human leukemia cells by regulation of COP1. J Cell Biochem.

[47] Shiraishi M, Shintani Y, Shintani Y, Ishida H, Saba R, Yamaguchi A (2016). Alternatively activated macrophages determine repair of the infarcted adult murine heart. J Clin Invest.

[48] Satoh T, Kidoya H, Naito H, Yamamoto M, Takemura N, Nakagawa K (2013). Critical role of Trib1 in differentiation of tissue-resident M2-like macrophages. Nature.

[49] Asimakopoulos F, Kim J, Denu RA, Hope C, Jensen JL, Ollar SJ (2013). Macrophages in multiple myeloma: emerging concepts and therapeutic implications. Leuk Lymphoma.

[50] Chen H, Li M, Sanchez E, Soof CM, Bujarski S, Ng N (2020). JAK1/2 pathway inhibition suppresses M2 polarization and overcomes resistance of myeloma to lenalidomide by reducing TRIB1, MUC1, CD44, CXCL12, and CXCR4 expression. Br J Haematol.

[51] Jurj A, Pop LA, Zanoaga O, Ciocan-Cartita CA, Cojocneanu R, Moldovan C (2020). New Insights in Gene Expression Alteration as Effect of Paclitaxel Drug Resistance in Triple Negative Breast Cancer Cells. Cell Physiol Biochem.

[52] Tang B, Wu W, Zhang Q, Sun Y, Cui Y, Wu F (2015). Inhibition of tribbles protein-1 attenuates radioresistance in human glioma cells. Sci Rep.

[53] Miyajima C, Inoue Y, Hayashi H (2015). Pseudokinase tribbles 1 (TRB1) negatively regulates tumor-suppressor activity of p53 through p53 deacetylation. Biol Pharm Bull.

[54] Mantovani A, Sica A (2010). Macrophages, innate immunity and cancer: balance, tolerance, and diversity. Curr Opin Immunol.

[55] Martinez FO, Helming L, Gordon S (2009). Alternative activation of macrophages: an immunologic functional perspective. Annu Rev Immunol.

[56] Wang F, Zhang S, Jeon R, Vuckovic I, Jiang X, Lerman A (2018). Interferon Gamma Induces Reversible Metabolic Reprogramming of M1 Macrophages to Sustain Cell Viability and Pro-Inflammatory Activity. EBioMedicine.

[57] Sica A, Mantovani A (2012). Macrophage plasticity and polarization: *in vivo* veritas. J Clin Invest.

[58] Miyajima C, Itoh Y, Inoue Y, Hayashi H (2015). Positive Regulation of Interleukin-2 Expression by a Pseudokinase, Tribbles 1, in Activated T Cells. Biol Pharm Bull.

[59] Dugast E, Kiss-Toth E, Docherty L, Danger R, Chesneau M, Pichard V (2013). Identification of tribbles-1 as a novel binding partner of Foxp3 in regulatory T cells. The Journal of biological chemistry.

[60] Liu YH, Tan KA, Morrison IW, Lamb JR, Argyle DJ (2013). Macrophage migration is controlled by Tribbles 1 through the interaction between C/EBPbeta and TNF-alpha. Vet Immunol Immunopathol.

[61] Ostertag A, Jones A, Rose AJ, Liebert M, Kleinsorg S, Reimann A (2010). Control of adipose tissue inflammation through TRB1. Diabetes.

[62] Angyal A, Kiss-Toth E (2012). The tribbles gene family and lipoprotein metabolism. Current opinion in lipidology.

[63] Danger R, Dugast E, Braza F, Conchon S, Brouard S (2015). Deciphering the role of TRIB1 in regulatory T-cells. Biochem Soc Trans.

[64] Saijou E, Enomoto Y, Matsuda M, Yuet-Yin Kok C, Akira S, Tanaka M (2018). Neutrophils alleviate fibrosis in the CCl4-induced mouse chronic liver injury model. Hepatology communications.

[65] Mack EA, Stein SJ, Rome KS, Xu L, Wertheim GB, Melo RCN (2019). Trib1 regulates eosinophil lineage commitment and identity by restraining the neutrophil program. Blood.

[66] Ying W, Fu W, Lee YS, Olefsky JM (2020). The role of macrophages in obesity-associated islet inflammation and beta-cell abnormalities. Nat Rev Endocrinol.

[67] Locati M, Curtale G, Mantovani A (2020). Diversity, Mechanisms, and Significance of Macrophage Plasticity. Annu Rev Pathol.

[68] van der Laan SW, Harshfield EL, Hemerich D, Stacey D, Wood AM, Asselbergs FW (2018). From lipid locus to drug target through human genomics. Cardiovasc Res.

[69] Sumegi K, Jaromi L, Magyari L, Kovesdi E, Duga B, Szalai R (2015). Functional variants of lipid level modifier MLXIPL, GCKR, GALNT2, CILP2, ANGPTL3 and TRIB1 genes in healthy Roma and Hungarian populations. Pathol Oncol Res.

[70] Bauer RC, Yenilmez BO, Rader DJ (2015). Tribbles-1: a novel regulator of hepatic lipid metabolism in humans. Biochem Soc Trans.

[71] Soubeyrand S, Martinuk A, Naing T, Lau P, McPherson R (2016). Role of Tribbles Pseudokinase 1 (TRIB1) in human hepatocyte metabolism. Biochim Biophys Acta.

[72] Makishima S, Boonvisut S, Ishizuka Y, Watanabe K, Nakayama K, Iwamoto S (2015). Sin3A-associated protein, 18 kDa, a novel binding partner of TRIB1, regulates MTTP expression. Journal of lipid research.

[73] Burkhardt R, Toh SA, Lagor WR, Birkeland A, Levin M, Li X (2010). Trib1 is a lipid- and myocardial infarction-associated gene that regulates hepatic lipogenesis and VLDL production in mice. J Clin Invest.

[74] Nagiec MM, Skepner AP, Negri J, Eichhorn M, Kuperwasser N, Comer E (2015). Modulators of hepatic lipoprotein metabolism identified in a search for small-molecule inducers of tribbles pseudokinase 1 expression. PLoS One.

[75] Softic S, Gupta MK, Wang GX, Fujisaka S, O'Neill BT, Rao TN (2017). Divergent effects of glucose and fructose on hepatic lipogenesis and insulin signaling. The Journal of clinical investigation.

[76] Iwamoto S, Boonvisut S, Makishima S, Ishizuka Y, Watanabe K, Nakayama K (2015). The role of TRIB1 in lipid metabolism; from genetics to pathways. Biochemical Society transactions.

[77] Kahali B, Halligan B, Speliotes EK (2015). Insights from Genome-Wide Association Analyses of Nonalcoholic Fatty Liver Disease. Semin Liver Dis.

[78] Woon PY, Kaisaki PJ, Braganca J, Bihoreau MT, Levy JC, Farrall M (2007). Aryl hydrocarbon receptor nuclear translocator-like (BMAL1) is associated with susceptibility to hypertension and type 2 diabetes. Proc Natl Acad Sci U S A.

[79] Marcheva B, Ramsey KM, Buhr ED, Kobayashi Y, Su H, Ko CH (2010). Disruption of the clock components CLOCK and BMAL1 leads to hypoinsulinaemia and diabetes. Nature.

[80] Ollila HM, Utge S, Kronholm E, Aho V, Van Leeuwen W, Silander K (2012). TRIB1 constitutes a molecular link between regulation of sleep and lipid metabolism in humans. Transl Psychiatry.

[81] Ma D, Liu T, Chang L, Rui C, Xiao Y, Li S (2015). The Liver Clock Controls Cholesterol Homeostasis through Trib1 Protein-mediated Regulation of PCSK9/Low Density Lipoprotein Receptor (LDLR) Axis. J Biol Chem.

[82] Uljon S, Xu X, Durzynska I, Stein S, Adelmant G, Marto JA (2016). Structural Basis for Substrate Selectivity of the E3 Ligase COP1. Structure.

[83] Holtkotte X, Dieterle S, Kokkelink L, Artz O, Leson L, Fittinghoff K (2016). Mutations in the N-terminal kinase-like domain of the repressor of photomorphogenesis SPA1 severely impair SPA1 function but not light responsiveness in Arabidopsis. The Plant journal: for cell and molecular biology.

[84] Kung JE, Jura N (2019). The pseudokinase TRIB1 toggles an intramolecular switch to regulate COP1 nuclear export. EMBO J.

[85] Grootaert MOJ, Moulis M, Roth L, Martinet W, Vindis C, Bennett MR (2018). Vascular smooth muscle cell death, autophagy and senescence in atherosclerosis. Cardiovascular research.

[86] Jaipersad AS, Lip GY, Silverman S, Shantsila E (2014). The role of monocytes in angiogenesis and atherosclerosis. Journal of the American College of Cardiology.

[87] Nordestgaard BG, Benn M, Schnohr P, Tybjaerg-Hansen A (2007). Nonfasting triglycerides and risk of myocardial infarction, ischemic heart disease, and death in men and women. JAMA.

[88] Varbo A, Benn M, Tybjaerg-Hansen A, Grande P, Nordestgaard BG (2011). TRIB1 and GCKR polymorphisms, lipid levels, and risk of ischemic heart disease in the general population. Arteriosclerosis, thrombosis, and vascular biology.

[89] Khallou-Laschet J, Varthaman A, Fornasa G, Compain C, Gaston AT, Clement M (2010). Macrophage plasticity in experimental atherosclerosis. PLoS One.

[90] Baitsch D, Bock HH, Engel T, Telgmann R, Muller-Tidow C, Varga G (2011). Apolipoprotein E induces antiinflammatory phenotype in macrophages. Arteriosclerosis, thrombosis, and vascular biology.

[91] Pan JX (2017). LncRNA H19 promotes atherosclerosis by regulating MAPK and NF-kB signaling pathway. European review for medical and pharmacological sciences.

[92] Reustle A, Torzewski M (2018). Role of p38 MAPK in Atherosclerosis and Aortic Valve Sclerosis. International journal of molecular sciences.

[93] Wang L, Jing J, Fu Q, Tang X, Su L, Wu S (2015). Association study of genetic variants at newly identified lipid gene TRIB1 with coronary heart disease in Chinese Han population. Lipids Health Dis.

[94] Manichaikul A, Palmas W, Rodriguez CJ, Peralta CA, Divers J, Guo X (2012). Population structure of Hispanics in the United States: the multi-ethnic study of atherosclerosis. PLoS Genet.

[95] Johnston JM, Angyal A, Bauer RC, Hamby S, Suvarna SK, Baidzajevas K (2019). Myeloid Tribbles 1 induces early atherosclerosis via enhanced foam cell expansion. Science advances.

[96] Ye Y, Wang G, Wang G, Zhuang J, He S, Song Y (2017). The Oncogenic Role of Tribbles 1 in Hepatocellular Carcinoma Is Mediated by a Feedback Loop Involving microRNA-23a and p53. Front Physiol.

[97] Li Y, Rong G, Kang H (2017). Taxotere-induced elevated expression of IL8 in carcinoma-associated fibroblasts of breast invasive ductal cancer. Oncol Lett.

[98] Wang Y, Wu N, Pang B, Tong D, Sun D, Sun H (2017). TRIB1 promotes colorectal cancer cell migration and invasion through activation MMP-2 via FAK/Src and ERK pathways. Oncotarget.

[99] Briffa R, Um I, Faratian D, Zhou Y, Turnbull AK, Langdon SP (2015). Multi-Scale Genomic, Transcriptomic and Proteomic Analysis of Colorectal Cancer Cell Lines to Identify Novel Biomarkers. PLoS One.

